# Mitochondria as Key Targets of Cardioprotection in Cardiac Ischemic Disease: Role of Thyroid Hormone Triiodothyronine

**DOI:** 10.3390/ijms16036312

**Published:** 2015-03-19

**Authors:** Francesca Forini, Giuseppina Nicolini, Giorgio Iervasi

**Affiliations:** 1 CNR Institute of Clinical Physiology, Via G. Moruzzi 1, Pisa 56124, Italy; E-Mails: nicolini@ifc.cnr.it (G.N.); iervasi@ifc.cnr.it (G.I.); 2 Tuscany Region G. Monasterio Foundation, Via G. Moruzzi 1, Pisa 56124, Italy

**Keywords:** cardiac ischemia/reperfusion injury, low T3 syndrome, mitochondrial dysfunction, cardioprotection

## Abstract

Ischemic heart disease is the major cause of mortality and morbidity worldwide. Early reperfusion after acute myocardial ischemia has reduced short-term mortality, but it is also responsible for additional myocardial damage, which in the long run favors adverse cardiac remodeling and heart failure evolution. A growing body of experimental and clinical evidence show that the mitochondrion is an essential end effector of ischemia/reperfusion injury and a major trigger of cell death in the acute ischemic phase (up to 48–72 h after the insult), the subacute phase (from 72 h to 7–10 days) and chronic stage (from 10–14 days to one month after the insult). As such, in recent years scientific efforts have focused on mitochondria as a target for cardioprotective strategies in ischemic heart disease and cardiomyopathy. The present review discusses recent advances in this field, with special emphasis on the emerging role of the biologically active thyroid hormone triiodothyronine (T3).

## 1. Introduction

Acute myocardial infarction (AMI) leading to ischemic heart disease is a major debilitating disease and important cause of death worldwide [1]. Deprivation of oxygen and nutrients following coronary occlusion is the primary cause of damage to the myocardium and its severity depends on the extent and duration of artery obstruction. Although timely, reperfusion effectively reduces short-term mortality, the reperfusion process itself yields additional injury, including cardiomyocyte dysfunction and death, which in the long run prompts adverse cardiac remodeling [1,2,3]. As a consequence, prevention or limitation of cardiac damage in the early stages of reperfusion is a crucial step in ameliorating patient prognosis.

Multiple lines of evidence show that mitochondrial functional impairments are critical determinants for myocyte loss during the acute ischemic stage, as well as for the progressive decline of surviving myocytes during the subacute and chronic stages [3,4,5,6]. Therefore, mitochondrial dysfunction is considered to be one of the major mechanisms in the pathogenesis of ischemia/reperfusion injury (IRI) and cardiomyopathy.

In spite of promising mitochondria-targeted therapeutic strategies emerging from experimental studies, very few have successfully completed clinical trials. As such, the mitochondrion is a potential untapped target for new therapies. Although ischemic pre-conditioning is a potent protective strategy first reported many decades ago [7], its utility in myocardial ischemia (MI) patients with an abrupt onset of disease undermines implementation of preconditioning in the clinical settings. Therefore, most modern approaches focus on the application of pharmacological or ischemic post-conditioning maneuvers to combat reperfusion injury and adverse cardiac remodeling [8].

Along this line, a growing body of clinical and experimental evidence shows that thyroid hormone (TH) supplementation may offer a novel option for cardiac diseases [9,10,11,12]. Indeed, 3,5,3'-triiodothyronine (T3), the biologically active form of TH, significantly declines after AMI both in animal models and in patients [13,14,15], with “low-T3 Syndrome” (low-T3S) being a strong independent prognostic predictor of death and major adverse cardiac events [16]. Consistently, treatment for low-T3S exerts cardioprotective effects in both humans and animal models [17,18,19,20].

Since the mitochondrion is a common effector of cardioprotective strategy and a main target of TH action [21,22,23], this review has a dual purpose: (1) to summarize the mitochondria-targeted noxious pathways and protective signaling that could be exploited to improve post-ischemic cardiac recovery and (2) to integrate classic and novel TH actions in a unified, mitochondria-centered picture that highlights how the crosstalk of TH with those molecular networks favors post-ischemic cardiomyocytes’ survival.

## 2. Triggers of Mitochondrial-Dependent Cardiomyocyte Death in Ischemia/Reperfusion

### 2.1. Mitochondrial Dysfunction in Ischemia/Reperfusion

A wide spectrum of metabolic and ionic derangements occur in ischemia/reperfusion (I/R), culminating in mitochondrial impairment. Oxygen deprivation during ischemia arrests oxidative phosphorylation, decreasing intracellular ATP and favoring anaerobic glycolysis. The accumulation of lactic acid decreases the intracellular pH. As a consequence, the Na^+^/H^+^ antiporter is activated in an attempt to restore the pH. The resulting accumulation of cytosolic sodium reverses the direction of the Na^+^/Ca^2+^ exchanger, leading to an increase in intracellular Ca^2+^ levels. The mitochondria act as a buffer for intracellular calcium, which ultimately causes calcium overload in the mitochondria [24,25]. This leads to an increase in ROS production from mitochondrial electron transfer complexes I and III, which consequently causes a decrease in anti-oxidant defenses [26,27,28]. The increased oxygen tension at the onset of reperfusion results in a greater burst of oxidative stress [29], which worsens mitochondrial dysfunction and alters membrane properties [5,30,31,32]. Damage to the mitochondrial outer membrane along with activation of the proapoptotic BCL-2 proteins leads to mitochondrial outer membrane permeabilization, release of cytochrome *c*, caspase activation, and apoptosis [33]. Massive oxidative stress can lead to a sudden increase in inner mitochondrial membrane permeability that is attributable to the opening of the so-called permeability transition pore (PTP). Opening of the PTP (PTPO) is accompanied by release of ROS and calcium [34,35]; this can propagate the damage to neighboring mitochondria and culminate in activation of calcium-dependent proteases (calpains) and lipases (cPLA2), inducing necrotic cell death [30,36]. The molecular nature of the PTP remains controversial, but current evidence implicates a matrix protein, Cyclophilin-D (Cyp-D), and two inner membrane proteins, adenine nucleotide translocase (ANT) and the phosphate carrier (PiC) [4,37,38,39].

An array of stress-responsive signaling pathways activated during early reoxygenation or in post-ischemic wound healing has been implicated in the regulation of these mitochondrial changes, and thus represents potential targets for therapeutic intervention.

### 2.2. The p38 Mitogen-Activated Protein Kinase Intracellular Signaling

A highly conserved component of myocyte stress-responsiveness in I/R involves signaling through a family of serine-threonine kinase effectors known as p38 mitogen-activated protein kinase (p38MAPK). Four separate p38MAPK isoforms, including p38α, p38β, p38γ, and p38δ, have been identified. Each p38 isoform phosphorylates a diverse array of intracellular proteins including stress-responsive transcription factors [40].

This signaling cascade ultimately converges in mitochondria to enhance oxidative stress and mitochondrial-dependent cardiomyocyte death [41,42,43]. Among the pro-apoptotic targets activated by p38Mapk in I/R, the tumor suppressor protein p53 and Bax play key roles in determining both acute cell injury and post-ischemic adverse remodeling [44,45,46]. Also, it has been demonstrated that p38 MAPK plays a causative role in the inhibition of the anti-apoptotic Bcl-2 protein [47]. Accordingly, inhibitors of p38 signaling have been shown to confer protection from IRI [48].

#### TH Inhibits p38MAPK under Stress Conditions

TH exhibits a prominent role in the regulation of p38MAPK. In the post-ischemic rat brain, thyroxine, T4, treatment was protective through its p38-targeted anti-apoptotic and anti-inflammatory mechanism [49]. In Langendorff-perfused rat heart models of I/R, long-term T4 pretreatment or acute T3 administration markedly improved post-ischemic recovery of left ventricular performance while reducing cardiomyocyte death markers and blunting the activation of p38MAPK [50,51]. As suggested by a subsequent study, this effect was mediated at least in part by the thyroid hormone receptor α1 (TRα1) [52]. Indeed, in a mouse model of AMI, pharmacological inhibition of TRα1 further depressed post-ischemic cardiac function and was accompanied by marked activation of p38MAPK [52]. 

### 2.3. Tumor Suppressor Protein p53

#### 2.3.1. p53 and Cardiomyocyte Death: Direct Action

Tumor suppressor protein p53 accumulates in the myocardium after myocardial infarction, and plays an important role in the progression to heart failure. It is well established that p53 can trigger apoptosis through the mitochondrial pathway [53]. For example, it can trans-activate Bax, the pro-apoptotic member of the BCL-2 family that translocates from the cytosol to mitochondria, causing the release of apoptotic proteins [54,55]. Besides its classic role, a broader role in organ homeostasis is just beginning to be understood. It has recently been reported that in response to oxidative stress, p530 accumulates in the mitochondrial matrix and triggers mitochondrial PTPO and necrosis by physical interaction with the PTP regulator Cyclophilin D (Cyp-D) [56]. p53 also plays a critical role in other important processes that regulate mitochondrial integrity but are impaired in I/R, such as mitochondrial morphology and mitophagy [57,58]. 

#### 2.3.2. p53 Regulation of Mitochondrial Morphology

Mitochondria change their morphology by undergoing either fusion or fission, resulting in either elongated, tubular, interconnected mitochondrial networks or fragmented, discontinuous mitochondria, respectively [59,60]. These two opposing processes are regulated by the mitochondrial fusion proteins: mitofusin (Mfn) 1, Mfn2, and optic atrophy protein 1(OPA1); and the mitochondrial fission proteins: dynamin-related protein 1 (Drp1) and human mitochondrial fission protein 1 (hFis1). The fine balance between mitochondrial fusion and fission within a cell may be upset by a variety of factors, including oxidative stress [61] and ischemia [34,62], which can predispose the cell to apoptosis and mitochondrial PTPO [63], critical mediators of IRI.

p53 affects the mitochondrial dynamic by two opposite mechanisms that disrupt the equilibrium between fission and fusion, promoting cell death. In one way, p53 may upregulate Drp1 with consequent activation of excessive mitochondrial fission [64]. Drp1, in turn, stabilizes p53 in the mitochondria to trigger necrosis [65]. On the other hand, p53 may promote indirect, Bax-mediated, excessive mitochondrial fusion leading to cell necrosis as well [66].

#### 2.3.3. p53 Effect on Mitophagy

In response to stress, cells have developed mitophagy, a defense mechanism that involves selective sequestration and subsequent degradation of the dysfunctional mitochondrion [67]. In I/R, mitophagy functions as an early cardioprotective response, favoring the removal of damaged mitochondria before they can cause activation of cell death [58]. The E3 ubiquitin ligase Parkin was recently discovered to play an important role in targeting damaged mitochondria for removal via autophagy in cardiomyocytes [58]. The proposed mechanism involves Mfn2 activation and Parkin recruitment from the cytosol to depolarized mitochondria [68]. Interestingly, another report showed Parkin localization to depolarized mitochondria even in the absence of Mfn2, which could indicate the presence of alternative mechanisms for Parkin translocation [69].

In the mouse heart, p53 cytosolic accumulation induces mitochondrial dysfunction by binding to Parkin and disturbing its translocation to damaged mitochondria and their subsequent clearance by mitophagy [70]. On the contrary, p53 knock-down preserved mitophagic flux under ischemia without a change in cardiac tissue ATP content [71]. Analysis of autophagic mediators acting downstream of p53 revealed that the TP53-induced glycolysis and apoptosis regulator (TIGAR) mediated the inhibition of myocyte mitophagy responsible for impairment of mitochondrial integrity and subsequent apoptosis, and this process is closely involved in p53-dependent ventricular remodeling after myocardial infarction [71].

## 3. Promoters of Mitochondria-Mediated Cardioprotection in Ischemia/Reperfusion

### 3.1. The Reperfusion Injury Salvage Pathway

A central biochemical pathway involved in cytoprotection is the phosphoinositide 3-kinase (PI3K) pathway, also known as the reperfusion injury salvage kinase (RISK) pathway. This pathway consists of a tyrosine kinase receptor (RTK) whose activation results in the recruitment of PI3K. Next, PI3K activates Akt, which in turn phosphorylates downstream kinases [72]. Regarding the heart, the literature on Akt is extensive [73] and has largely established Akt as a key pro-survival kinase in normal cardiac homeostasis and in response to injury. Classically, Akt activation promotes survival via inhibition of pro-apoptotic Bcl-2 family proteins Bax and Bad, limiting mitochondrial outer membrane (OMM) permeabilization and thereby blocking release of cytochrome *c* and caspase-mediated apoptosis. The RISK pathway is also implicated in PTP regulation and preservation of mitochondrial membrane potential (ΔΨm) [74]. The glycogen synthase kinase 3-β (GSK-3β) is a key downstream target of Akt and is inactive when phosphorylated. Thus, GSK-3β phosphorylation by Akt or other upstream mediators results in inhibition of GSK-3β-activated targets. For example, inactivation of GSK-3β by Akt reduces mitochondrial Bax recruitment [75] as well as PTPO [76,77]. Enhancement of p53 activity by GSK-3β and GSK-3β interaction with CypD may have a role in mPTP opening [78,79]. The use of GSK-3β inhibitors in the post-ischemic setting is hampered by the side effect of inhibiting the physiological function of GSK-3β [80]. To overcome this limitation, selective inhibition of GSK-3β mitochondria uptake has been reported as a promising and novel approach to cardioprotection from lethal reperfusion injury [81].

The recruitment of the RISK pathway also induces phosphorylation-dependent activation of the endothelial nitric oxide synthase (eNOS), which is expected to block PTPO through its release of nitric oxide (NO) [82]. In turn, NO triggers the opening of the mitochondrial ATP-dependent potassium channels (mitoKATP) [83], a cardioprotective process that has been causally related to post-conditioning [84]. Furthermore, an increase in NO availability may enhance mitochondrial protein *S*-nitrosylation (SNO) and promote cardioprotection [85,86]. Finally, the RISK pathway has also been shown to confer cardioprotection against IRI by modulating Mfn1-dependent mitochondrial morphology [86,87].

#### Role of Thyroid Hormone in the Activation of Reperfusion Injury Salvage Pathway

THs are critically involved in the activation of the RISK pathways in both physiological and stress conditions. Rapid T3-mediated activation of PI3K by cytosolic TRα1, and subsequent activation of the Akt-mTOR signaling pathway, has been proposed as one of the mechanisms by which TH regulates physiological cardiac growth [88]. T3 administration can prevent serum starvation-induced neonatal cardiomyocyte apoptosis via Akt [89]. *In vivo* T4 treatment has been shown to cause phosphorylation of Akt and downstream signaling targets such as GSK-3β and mTOR in rat heart ventricles [90]. The Akt-mediated cardioprotective action of TH was confirmed in an experimental model of rat myocardial ischemia, where early short-term treatment of T3 reduced myocytes apoptosis through activation of Akt [19]. In a recent study, TH was found to have a dose-dependent effect on Akt phosphorylation, which may be of physiological relevance [91]. Mild activation of Akt caused by the replacement dose of TH resulted in favorable effects, while further induction of Akt signaling by higher doses of TH was accompanied by increased mortality and activation of extracellular signal-regulated kinases (ERK), some of the most well-studied kinases in relation to pathological remodeling [92]. This study may be of important therapeutic relevance because it shows that TH replacement therapy may be sufficient to restore cardiac function, while excessive TH doses may be detrimental rather than beneficial.

### 3.2. Inhibition of p53 Signaling

Given the detrimental effects of p53 in the myocardial IRI, this molecule may be proposed as a central hub in stress-induced apoptosis and necrosis instigated in mitochondria and may act as a novel therapeutic target [93]. 

#### Role of Thyroid Hormone in the Inhibition of p53 Signaling

Thyroid hormone is a critical regulator of p53 activity. A p53-centered anti-apoptotic action of TH has been well characterized in tumor cells [94,95]. In a rat model of post-ischemic acute stroke, TH treatment reduced cerebral infarction while limiting cell death through modulation of the p53 targets Bax and BCl2 [49]. On the other hand, p53 is able to hamper TH signaling. Early studies showed that the physical interaction of thyroid hormone receptors (TRs) with p53 inhibited the binding of TRs to the TH-responsive elements (TREs) in a concentration-dependent manner and that this interaction negatively regulated the TRs’ signaling pathways [96,97]. Although these data collectively suggest that the cross-talk between p53 and TH may play an important role in physiological and pathological conditions, its role in cardiac disease evolution is only beginning to be explored. A recent paper reported a critical role for TH in inhibiting the p53-dependent activation of mitochondrial-mediated cell death in a model of cardiac I/R [98]. In this study, the low-T3S following the ischemic insult was accompanied by an up-regulation of p53 and activation of its downstream events, such as Bax induction and mitochondrial impairment. Early T3 administration at near-physiological dose improved the recovery of post-ischemic cardiac performance. At the molecular level, T3 blunted p53 and Bax up-regulation in the area at risk (AAR), thus preserving mitochondrial function and decreasing apoptosis and necrosis extent in the AAR [98]. Similarly, in cardiomyocytes exposed to oxidative stress, T3 treatment reduced cell death, preserved mitochondrial biogenesis and membrane potential, and limited p53 upregulation [98,99]. 

### 3.3. Targeting Mitochondrial Oxidative Stress

In accordance with a role for mitochondrial dysfunction and ROS production in the pathogenesis of heart disease, it has been shown that targeted mitochondrial ROS scavenging reduces remodeling whereas non-targeted ROS treatment has no effect [32]. This is in agreement with the lack of benefit provided by non-targeted anti-oxidants in the clinical arena [100,101], and supports the general concept of compartmentalized signaling. This concept implies close vicinity of signaling molecules to provide local control over second messengers; in this regard, targeted ROS scavenging, which accumulates manifold at the microdomains of ROS formation in mitochondria, might be more efficient in the specific targeting of cellular ROS signaling. One relevant paper suggests that mitochondria-targeted Bendavia may be extremely effective in preventing reperfusion-induced damage to cardiac mitochondria [102]. Mitoquinone (mitoQ), a coenzyme Q analog, easily crosses phospholipid bilayers and is driven to concentrate within mitochondria by the large electrochemical membrane potential. The respiratory chain reduces mitoQ to its active ubiquinol antioxidant form to limit myocardial I/R injury [103]. The SS-31 (Szeto-Schiller) peptide is also of interest since it is cell-permeable and specifically targeted to inner mitochondrial membranes based on its residue sequence, with an anti-oxidant dimethyltyrosine moiety. SS-31 has been shown to be taken up by the heart in an *ex vivo* reperfusion system and was protective against I/R injury [104]. The peptides SS-02 and SS-31 were also protective against cardiac I/R injury when added during reperfusion [105].

#### Role of Thyroid Hormone in the Inhibition of Mitochondrial Oxidative Stress

It has recently been shown that TH has a mitochondria-targeted antioxidant protective effect under *in vitro* stress conditions and after myocardial infarction *in vivo* [98,99,106]. In cultured cardiomyocytes, T3 treatment decreased oxidative stress-induced cell death while maintaining mitochondrial function [99]. These effects were prevented by inhibitors of mitoKATP channel opening, suggesting that activation of the mitoKATP channel in rescued mitochondria is an important protective mechanism elicited by T3 against oxidative stress-mediated cell death [99]. In a post-ischemic HF model, TH administration during the post-infarction period leads to normalization of the myocardial performance index, reduction of ROS level, and stimulation of cytosolic and mitochondrial anti-oxidant defenses [106]. In an experimental AMI model, T3 supplementation reduced mitochondrial superoxide production and limited inner mitochondrial membrane depolarization, thus improving mitochondrial function and cell viability [99].

### 3.4. Inhibition of Mitochondrial Permeability Transition Pore Opening

Since the initial reports on the existence of mitochondrial cyclophilin in the late 1980s, a vast majority of studies have recognized the crucial role of Cyp-D in PTP regulation. In animal models, inhibition of Cyp-D by either pharmacologic targeting [107,108], genetic ablation [109,110], or RNA interference [111], provides strong protection from both reperfusion injury and post ischemic HF. Although chronic pharmacological inhibition of CypD has been shown to cause metabolic reprogramming and worsening of pressure-induced HF [112], its acute inhibition to attenuate lethal IR injury holds great promise for reducing myocardial infarct size in humans [8,113]. Besides Cyp-D, several physiological regulators of mPTP function may be exploited to confer cardioprotection from I/R injury.

One important class of endogenous transducers of cell stress signals are the signal transducer and activator of transcription (STAT) proteins. Several STAT isoforms are expressed in the heart; among them, STAT3 is involved in the reduction of post-ischemic myocardial injury [114]. The infarct size reduction by ischemic post-conditioning is also attenuated in STAT3-KO mice [115]. STAT3 has been localized in mitochondria, where it contributes to cardioprotection by stimulating respiration and inhibiting the Ca^2+^-induced mitochondrial PTPO [116].

Nitric oxide (NO) is another important signaling molecule that has been shown to reduce myocardial injury in a number of ischemia/reperfusion models. For example, brief periods of NO breathing reduced myocardial injury from ischemia/reperfusion in mice and pigs [117,118,119]. A critical process during NO-induced cardioprotection is to prevent mitochondrial PTPO potentially via targeting of the ANT component of the pore-forming complex [120].

Hypoxia inducible factor 1 α (HIF-1α) is an oxygen-sensitive transcription factor that enables aerobic organisms to adapt to hypoxia. This is achieved through the transcriptional activation of up to 200 genes, many of which are critical to cell survival [121]. Under normoxic conditions, the hydroxylation of HIF-1α by prolyl hydroxylase domain-containing (PHD) enzymes targets it for proteosomal degradation. However, under hypoxic conditions, PHD activity is inhibited, thereby allowing HIF-1α to accumulate and translocate to the nucleus, where it binds to the hypoxia-responsive element sequences of target gene promoters. Experimental studies suggest that stabilization of HIF-1α may protect the heart against the detrimental effects of acute I/R injury [121]. 

#### Role of TH in the Inhibition of Permeability Transition Pore Opening

The mechanisms underlying the myocyte-directed protective effect of HIF are not completely clear. A recent paper showed that HIF-1α stabilization, by either a pharmacological or genetic approach, protected the heart against acute IRI by inhibiting mitochondrial PTPO and decreasing mitochondrial oxidative stress [122]. Accordingly, T3 replacement has been shown to induce HIF-1α stabilization in a post-ischemic HF model, which was related to better preserved mitochondrial activity and cardiac performance [99].

### 3.5. Mitochondrial Biogenesis

Mitochondrial biogenesis has emerged as an important point in the multi-site control of mitochondrial function and a putative target for therapeutic intervention against cardiac IRI [123,124]. Mitochondrial biogenesis includes regulation of mitochondrial protein expression, their assembly within the mitochondrial network, and replication of mitochondrial DNA (mtDNA). Of the 1500 proteins representing the mitochondrial proteome, mtDNA provides 13 subunits of the oxidative phosphorylation system together with ribosomal and transfer RNAs; whereas more than 98% of the mitochondrial protein requirement is encoded by the nuclear genome [125]. Hence, a spatial and temporal coordination of nuclear and mitochondrial genomes is necessary to ensure that all mitochondrial components are available for correct assembly. The master regulator of the process is the nuclear-encoded peroxisome proliferator-activated receptor-γ coactivator-1α (PGC1-α). PGC-1α lacks DNA-binding activity but interacts and coactivates numerous transcription factors driving mitochondrial biogenesis, energy metabolism, fatty acid oxidation, and antioxidant activity [126]. In particular, PGC-1α activates nuclear transcription factors (NTFs) leading to upregulation of nuclear-encoded proteins. Nuclear-encoded proteins are imported into mitochondria through the outer-membrane (TOM) or inner-membrane (TIM) translocase transport machinery. Finally, nuclear- and mitochondrial-encoded subunits of the respiratory chain are assembled [127]. A downregulation of the entire pathway of mitochondrial biogenesis was reported both in AMI and in HF evolution [128,129]. Reduced PGC-1α activity and gene expression have been observed in several experimental models of pathologic cardiac hypertrophy, and HF [130,131] and has been involved in the pathogenesis of human heart disease [132]. It has been shown that in the heart, pathological stressors such as ischemia are associated with a downregulation of mitochondrial biogenesis via PGC-1α activity [133], and that impairment of the PGC-1α-mediated mitochondrial biogenesis increased heart vulnerability to IRI [134]. Accordingly, upregulation of the PGC-1α pathway confers protection against simulated I/R in cardiomyoblast cells [135]. Moreover, the induction of PGC-1α protein upregulates a broad spectrum of ROS detoxification systems, such as superoxide dismutase 2 (SOD2) and glutathione peroxidase-1 [136]. Hence, a putative mechanism whereby the mitochondrial biogenesis program may additionally augment tolerance to cardiac ischemia is via ROS detoxification.

During mitochondrial biogenesis, the coordinated transcription and replication of the mitochondrial genome is carried out via the nuclear-encoded mitochondrial transcription factor A (mtTFA), a downstream effector of PGC1-α signaling. It has long been recognized that post-ischemic adverse remodeling is frequently associated with qualitative and quantitative defects in mtDNA [137,138,139], and that a decline in mitochondrial function and mtDNA copy number play a major role in the development of post-ischemic heart disease [31,140]. In accordance, targeted disruption of mtTFA specifically within cardiac tissue resulted in a significant decrease in electron transport capacity, spontaneous cardiomyopathy, and cardiac disease [141,142]. Conversely, increasing the expression of mtTFA within cardiac tissue offered protection from adverse remodeling induced by myocardial infarction [143].

Given the causative role of mitochondrial dis-homeostasis in adverse cardiac remodeling, understanding the stimuli, signals, and transducers that govern mitochondrial biogenesis pathways may have critical significance in the treatment of ischemic cardiovascular disorders. In the last few years, the NAD^+^-dependent protein deacetylase sirtuin 1 (SIRT1) has emerged as an important regulator of mitochondrial biogenesis [144,145]. Besides its epigenetic role in silencing of transcription by heterochromatin formation through histones modification, SIRT1 influences the activity of PGC-1α through a functional protein–protein interaction [146]. SIRT1 activation of PGC-1α enhances mitochondrial biogenesis, optimizes mitochondrial surface/volume ratio to reduce ROS production, and mounts an antioxidant defense [146,147]. Several lines of evidence show that SIRT1 has pivotal roles in cardiovascular function. Transgenic mice that overexpress SIRT1 in the heart are resistant to oxidative stress-related cardiac hypertrophy and ischemia/reperfusion injury [148,149]. In addition, the putative SIRT1 activator, resveratrol, a recognized mediator of mitochondrial biogenesis, can ameliorate heart ischemia or reperfusion injury, improve vascular functions, and ameliorate Ang II-induced cardiac remodeling [150,151].

#### Thyroid Hormone Is Key Regulator of Mitochondrial Biogenesis

Thyroid hormone plays a crucial role in regulation of mitochondrial biogenesis in both physiological and pathological conditions [24]. PGC-1α is rapidly and strongly induced by TH. PGC-1α expression and protein levels are increased 6 h after administration of T3, and this action is mediated by a TH responsive element (TRE) in the promoter [152,153,154]. In a rat model of post-ischemic HF, a low-T3S correlated with PGC-1α and mtTFA downregulation, which corresponded to decreased mitochondrial function in the border zone; T3 replacement rescued myocardial contractility and hemodynamic parameters, while maintaining the expression of PGC-1α and mtTFA and mitochondrial function [99]. Since the PGC-1α pathway is downregulated by p53 activation under oxidative stress conditions, the inhibitory role of T3 on p53 expression may be part of an additional and indirect mechanism by which TH controls PGC1-α levels in the post-cardiac ischemia setting [155].

The reduced PGC1-α level in the post-ischemic low-T3S is consistent with the activation of a fetal metabolic pathway observed in cardiomyopathy that is characterized by a preference for glucose over fat as a substrate for oxidative phosphorylation. Although such changes lower the oxygen consumed per ATP produced, the yield of ATP per substrate also decreases. Such inefficient metabolism lowers ATP and phosphocreatine levels and decreases metabolic reserve and flexibility, leading to pump dysfunction [156,157]. Therefore, T3 supplementation in low T3 post-ischemic cardiomyopathy may favor the normal mitochondrial homeostasis and metabolic flexibility of the heart, preventing adverse cardiac remodeling and HF evolution.

Thyroid-stimulated mitochondrial biogenesis appears to be mediated via specific TRs located in both the nuclear and mitochondrial compartments [21,22]. Wrutniak-Cabello *et al.* [158] reported the discovery in mitochondria of two *N*-terminally truncated forms of the T3 nuclear receptor, TRα1, with molecular weights of 43 and 28 kDa, respectively. While the function of p28 remains unknown, p43 is a T3-dependent transcription factor of the mitochondrial genome, acting through dimeric complexes involving at least two other truncated forms of nuclear receptors, mitochondria retinoid X receptor (mtRXR) and mitochondrial peroxisome proliferator-activated receptor (mtPPAR); p43 activation by T3 stimulates mitochondrial protein synthesis, respiratory chain activity, and mitochondriogenesis [23]. Similarly, Saelim *et al.* [159] reported that T3 bound TH truncated receptor isoforms (TRs) target mitochondria where they modulate inositol 1,4,5 trisphosphate (IP3)-mediated Ca^2+^ signaling [160] to inhibit apoptotic potency.

### 3.6. MicroRNAs

MicroRNAs (miRNAs) are a subset of regulatory molecules involved in several cellular processes of cardiac remodeling and heart failure (HF) [161,162,163,164], and have become an intriguing target for therapeutic interventions [165]. In response to diverse cardiac stresses such as myocardial I/R, miRNAs are reported to be up- or downregulated [166,167,168,169]. Some of them have recently attracted attention as regulators of mitochondrial function and mitochondrial cell death signaling in both myocardial I/R and *in vitro* models of oxidative stress [170,171,172,173,174]. 

#### Role of Thyroid Hormone in the Regulation of Cardioprotective miRNA

The miR-30 family members are abundantly expressed in the mature heart, but they are significantly downregulated in experimental I/R and *in vitro* after oxidative stress [174,175,176]. Li *et al.* [173] report that miR-30 family members are able to regulate apoptosis by targeting the mitochondrial fission machinery. In exploring the underlying molecular mechanism, they identified that miR-30 family members inhibited mitochondrial fission by suppressing the expression of p53 and its downstream target, dynamin-related protein 1 (Drp1) [174]. Therefore, maintenance of miR-30 levels in I/R may be regarded as cardioprotective. In a rat model of I/R, early short-term T3 supplementation at near-physiological dose maintained the post-ischemic level of miR-30a, leading to a depression of p53 and inhibition of p53 detrimental effects on mitochondria [98]. In turn, p53 is responsible for the post-ischemic inhibition of miR-499, another highly expressed cardiac miRNA with a key role in the regulation of mitochondrial dynamic [64]. MiR-499 levels are reduced in experimental ischemia as well as in anoxic cardiomyocytes, and this reduction is causally linked to apoptosis and the severity of myocardial infarction and cardiac dysfunction induced by I/R [64]. MiR-499 inhibits cardiomyocyte apoptosis through its suppression of calcineurin-mediated dephosphorylation of Drp1, thereby decreasing Drp1 accumulation in mitochondria and Drp1-mediated activation of the mitochondrial fission program [64].

## 4. Closing Remarks and Conclusions

**Figure 1:**
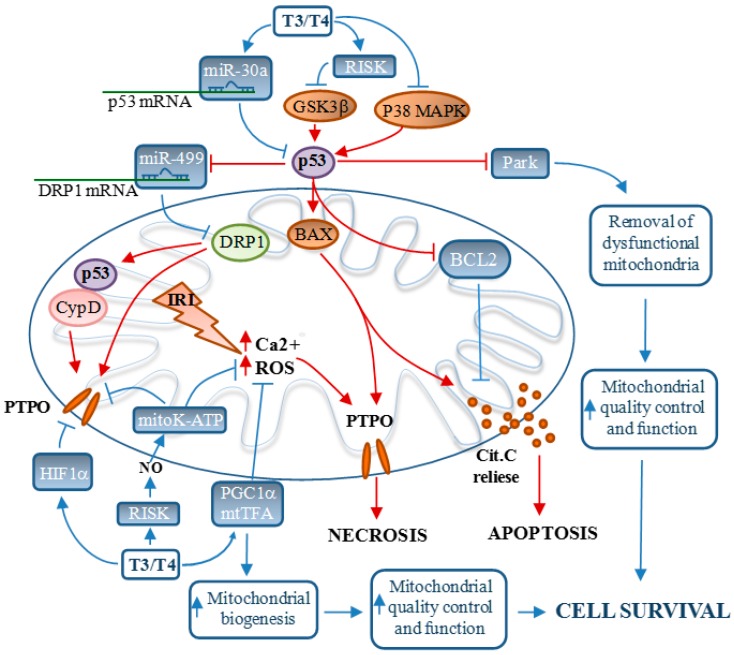
Schematic overview of the role of TH in the modulation of the mitochondrial pro-survival (blue connectors) or pro-death (red connectors) signaling networks that control cardiomyocyte fate in the I/R heart. CypD = Cyclophilin D; DRP1 = dynamin-related protein 1; GSK3β = glycogen synthase kinase 3-β; HIF1α = Hypoxia inducible factor 1 α, IRI = ischemia/reperfusion injuries; mitoK-ATP = mitochondrial ATP-dependent potassium channel; mtTFA = mitochondrial transcription factor A; Park = parkin; PTPO = permeability transition pore opening; PGC1-α = peroxisome proliferator-activated receptor-γ coactivator-1α; RISK = reperfusion injury salvage kinase.

In the last three decades, several biochemical pathways conveying I/R deleterious effects to the mitochondria have been characterized. In parallel, several endogenous protective molecules that enhance mitochondrial survival have been identified and, consequently, prevent progression to HF. As depicted in Figure 1, TH acts on both noxious and beneficial pathways to induce cardioprotection from IRI. Therefore, treatment of post-ischemic low-T3S appears to be a promising modality for reducing mitochondrial-driven IRI and preventing progression to HF. However, from a translational point of view, there are still some unsolved issues regarding the dose and timing of TH administration after AMI. To complicate the picture, a low-T3S in the very first hours after AMI is considered protective since it lowers the energetic demand and predisposes the heart to regenerative repair [177]. On the other hand, previous studies have demonstrated that post-MI LV remodeling, a major determinant of morbidity and mortality in overt HF [178,179], is an early process. As a consequence, it is expected that an efficacious intervention aimed at preventing the initial stages of remodeling would better contrast the progression towards HF [176]. In accordance, Henderson *et al.* [18] showed that L-T3 replacement, initiated one week after MI, improved ventricular performance without reversing cardiac remodeling. On the other hand, early T3 replacement limited post-ischemic cardiomyocyte loss and blunted adverse cardiac remodeling [19,98,99]. With TH administration, it is also critical to choose the right dose in order to limit cardiac remodeling and avoid the potentially adverse systemic effects (*i.e.*, thyrotoxicosis). In a previous study, an immediate long-term, but not controlled, supplementation of TH at a high dose in post-MI improved LV function and prevented cardiac remodeling, but also induced a thyrotoxic state [20], which in the long run may lead to heart dysfunction. These results were confirmed in a successive study demonstrating the dose-dependent bimodal effects of TH administration [91].

In conclusion, if we exclude the hyperacute post-MI period, the available evidence suggests that TH should be administered at a physiological or near-physiological dose in the early phase of the post-ischemic wound healing following reactivation of the endogenous regenerative process in order to obtain the maximal protective effect.

## Abbreviations

AMI: acute myocardial infarction
CypD: Cyclophilin D
DRP1: dynamin-related protein
GSK3β: glycogen synthase kinase 3-β
HIF1α: Hypoxia inducible factor 1 α
IRI: ischemia/reperfusion injuries
Low T3 syndrome: Low-T3S
mitoK-ATP: mitochondrial ATP-dependent potassium channel
mtTFA: mitochondrial transcription factor A
Park: parkin
PGC1-α: peroxisome proliferator-activated receptor-γ coactivator-1α
PTPO: permeability transition pore opening
P38MAPK: P38 mitogen activated protein kinase
RISK: reperfusion injury salvage kinase
